# Declined ELABELA plasma levels in hypertension patients with atrial fibrillation: a case control study

**DOI:** 10.1186/s12872-021-02197-x

**Published:** 2021-08-12

**Authors:** Zheng Ma, Lei Zhao, Ye-ping Zhang, Jiu-chang Zhong, Xin-chun Yang

**Affiliations:** 1 Department of Cardiology, Beijing Tongren Hospital, Capital Medical University, No. 1 Dongjiao Minxiang, Dongcheng District, Beijing, 100730 China; 2 Heart Center and Beijing Key Laboratory of Hypertension, Beijing Chaoyang Hospital, Capital Medical University, Beijing, 100020 China

**Keywords:** ELABELA, Hypertension, Atrial fibrillation, Correlative factor

## Abstract

**Background:**

Atrial fibrillation (AF) is a common arrhythmia in patients with hypertension. ELABELA, which has cardioprotective effects, is decreased in the plasma of patients with hypertension and might be associated with AF in the hypertensive population. This study aims to measure the ELABELA plasma levels in hypertension patients with and without AF and to analyse the related factors.

**Methods:**

A total of 162 hypertension patients with or without AF were recruited for our monocentric observational study. Subjects were excluded if they had a history of valvular heart disease, rheumatic heart disease, cardiomyopathy, thyroid diseases, or heart failure. The patients’ histories were recorded, and laboratory examinations were conducted. Plasma ELABELA was detected by immunoassay. Echocardiographs were performed, and parameters were collected by two experienced doctors. Binary logistic regression analysis was used to identify the association between ELABELA plasma level and AF in patients with hypertension.

**Results:**

Plasma ELABELA levels were lower in hypertension patients with AF than in those without AF (2.0 [1.5, 2.8] vs. 4.0 [3.4, 5.0] ng/ml, P < 0.001). ELABELA levels were correlated with age, heart rate, BNP levels and left atrial dimension. In addition to the left atrial dimension, ELABELA plasma levels were associated with AF in patients with hypertension (OR 0.081, 95% CI 0.029–0.224, P < 0.001). ELABELA levels were further decreased in the persistent AF subgroup compared with the paroxysmal AF subgroup (1.8 [1.4, 2.5] vs. 2.2 [1.8, 3.0] ng/ml, P = 0.012) and correlated with HR, BNP and ESR levels.

**Conclusions:**

ELALABELA levels were decreased in hypertension patients with AF and further lowered in the persistent AF subgroup. Decreased ELABELA plasma levels were associated with AF in hypertension patients and may be an underlying risk factor.

**Supplementary Information:**

The online version contains supplementary material available at 10.1186/s12872-021-02197-x.

## Background

Atrial fibrillation (AF) is a common arrythmia in patients with hypertension worldwide [1]. One-fifth of the risk factors for AF are attributable to hypertension, and the odds ratio for the development of AF in the hypertensive population is 1.4–1.5, revealing a close relationship between hypertension and AF [2]. Hypertension is an independent risk factor for thrombosis and bleeding in AF patients, making the prognosis of AF unfavourable [3, 4]. Additionally, effective blood pressure control using antihypertensive drugs (such as angiotensin converting enzyme inhibitors and angiotensin receptor blockers) can prevent AF development and recurrence [5]. Based on the fact that therapeutic interventions targeting AF risk factors have been shown to improve outcomes, it is important to identify hypertension patients at high risk of AF [6].

The apelin receptor (APJ) was regarded as an orphan G-protein-coupled receptor when apelin/APJ signalling was first reported in 1998. Apelin/APJ signalling is widely distributed in various tissues and plays key roles in embryonic development, tumour progression and cardiovascular disease prevention [7]. ELABELA, a novel endogenous ligand of APJ, was discovered in zebrafish embryos and promotes gastrulation movement [8]. ELABELA-32, the mature secretory form of ELABELA, has been detected in stem cells, the kidneys, the prostate, the vascular endothelium and plasma, and has been demonstrated to have vital cardioprotective effects in cardiovascular illnesses [9]. As reported, ELABELA plasma levels were reduced in hypertension patients [10], and those with low levels of ELABELA tended to develop hypertension [6]. Additionally, experimental evidence has revealed that ELABELA has positive inotropic action and plays anti-remodelling, anti-inflammatory and anti-fibrotic roles in the cardiovascular system [9], helping to attenuate the effects of risk factors for AF development including atrial enlargement, myocardial fibrosis and inflammatory response, and to reduce the incidence and burden of AF. Unfortunately, there has been no clinical study investigating the correlation between ELABELA plasma levels and AF in patients with hypertension.

In our study, we calculated the plasma level of ELABELA in hypertension patients with and without AF, and investigated the correlative clinical factors of ELABELA to identify the underlying risk factors for AF in hypertension patients.

## Methods

### Study population

Hypertension patients with and without AF were recruited continuously in our center from November 2018 to October 2019. The study protocol was approved by the ethics committee of the hospital, and all of the participants provided informed consent. Finally, a total of 81 hypertension patients with AF, including 45 patients with paroxysmal AF and 36 patients with persistent AF, were enrolled in the hypertension and AF group (HT + AF group). Another 81 age- and sex-matched hypertension patients without AF were enrolled in the hypertension group (HT group). Criteria for the diagnosis of hypertension and AF referred to the 2018 ESC/ESH guidelines for the management of arterial hypertension and the 2016 ESC guidelines for the management of atrial fibrillation, developed in collaboration with EACTS [11, 12]. The exclusion criteria were as follows: (1) pregnancy; (2) valvular heart disease; (3) rheumatic heart disease; (4) thyroid diseases; (5) heart failure; (6) severe renal dysfunction with an estimated glomerular filtration rate (eGFR) ≤ 30 ml/min/1.73 m^2^ at baseline; (7) tumours; and (8) severe infection. The study was approved by the local ethics committee of our faculty of medicine.

### Study process

Medical history and vital signs were collected at the time of enrollment. All blood samples were collected from peripheral vein fasting for at least 8 h in the morning. Brain natriuretic peptide (BNP), serum creatinine, haemoglobin A1C, serum lipids, the erythrocyte sedimentation rate (ESR), high sensitivity C-reactive protein, troponin I and homocysteine were tested in our clinical laboratory center according to standard operation procedures. The left atrial diameter (LAD), the left ventricular end-diastolic dimension, the left ventricular end-systolic dimension and the left ventricular ejection fraction were obtained by two experienced physicians with an EPIQ 7C echocardiography system (Philips).

### ELABELA enzyme immunoassay

Venous blood was drawn from an antecubital vein between 7:00 AM and 8:00 AM. Next, the sample was processed with centrifugation at 4 °C and 3000 rpm for 10 min within an hour. The plasma was separated and stored at − 80 °C in a 1.5 ml microcentrifuge tube until use. The plasma levels of ELABELA were tested with a commercialised human ELABELA ELISA Kit (Peninsula Laboratories International S-1508, Inc. USA). The operation procedures followed the instructions of the ELISA kit.

### Statistical analysis

Continuous data were presented as the mean ± SD or median (IQR). Categorical data were presented as numbers and percentages. The Mann–Whitney U test for continuous variables and Student’s t-test for normally distributed data with equal variances were used to compare the groups. Fisher’s exact test was employed to compare the categorical variables between the groups. Spearman’s correlation analyses were used to assess the relationship between ELABELA levels and the variables. The clinical characteristics associated with AF were analysed through binary logistic regression in all subjects. Further, the clinical characteristics associated with persistent AF were also examined through binary logistic regression in patients with hypertension and AF. Only variables that had P < 0.10 in the single-factor analysis were included in the multivariable model. All statistical analyses were performed using SPSS software version 23 (IBM Corporation, Armonk, NY). A p-value < 0.05 was considered statistically significant.

## Results

### Declined ELABELA levels in hypertension patients with AF

**Fig. 1:**
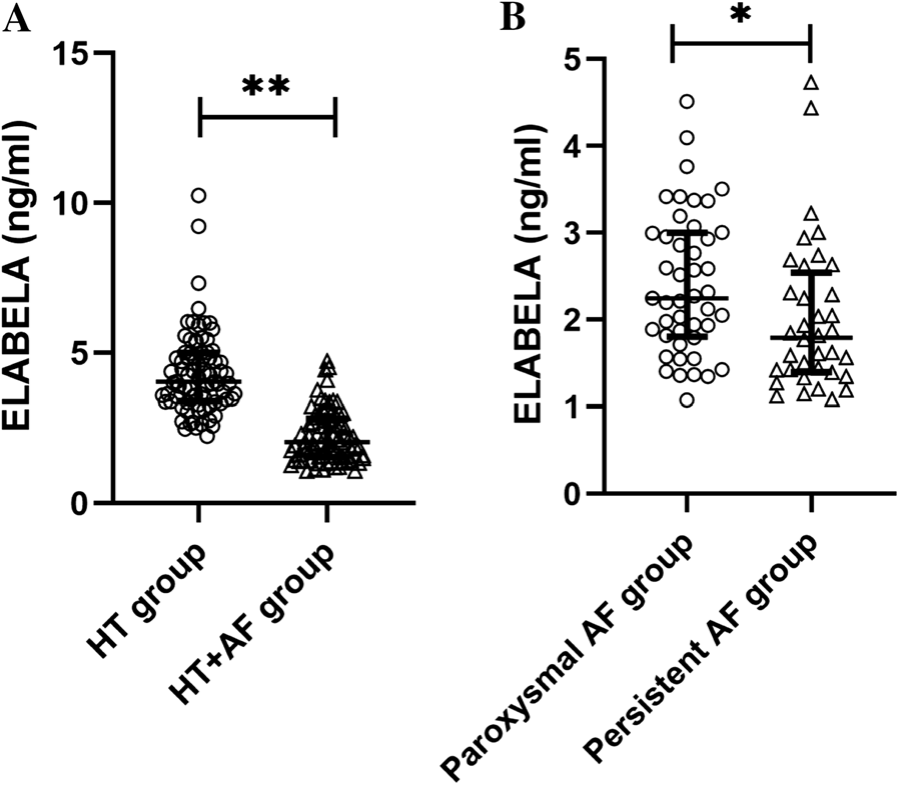
The ELABELA plasma levels in patients with hypertension and atrial fibrillation. **a** ELABELA plasma levels in hypertensive patients with or without atrial fibrillation; **b** ELABELA plasma levels in hypertensive patients with paroxysmal and persistent atrial fibrillation

A total of 162 hypertension patients with and without AF were enrolled in the HT group (n = 81, 64.4 ± 11.1 years) and the HT + AF group (n = 81, 66.9 ± 10.1 years), respectively; the other clinical characteristics are shown in Table 1. There were no significant differences in sex, body mass index, medicine history or blood pressure between the two groups. Patients without AF had a lower heart rate than patients with AF (74.2 ± 11.7 vs. 80.3 ± 14.3, P = 0.004). BNP levels in the HT group were much lower than those in the HT + AF group (26.5 [11.0, 53.3] vs. 106 [56.0, 183.5] pg/ml, P < 0.001). Higher ELABELA levels were observed in the HT group than in the HF + AF group (4.0 [3.4, 5.0] vs. 2.0 [1.5, 2.8] ng/ml, P < 0.001) (Fig. 1a). Other laboratory tests were similar in the two groups. Echocardiographic data revealed that the mean value of LAD was much larger in the HT + AF group than in the HT group (36.6 ± 5.3 vs. 42.6 ± 6.6 mm, P < 0.001), while the mean ventricular size was similar. Ventricular systolic function was better in the HT group than in the HT + AF group (66.2 ± 7.2 vs. 63.5 ± 8.1, P = 0.038).

**Table 1:** Comparison of the demographic and baseline characteristics of the hypertension patients with and without atrial fibrillation

	Total (n = 162)	HT group (n = 81)	HT + AF group (n = 81)	P-value
Age, years	65.7 ± 10.7	64.4 ± 11.1	66.9 ± 10.1	0.138
Male sex	46/81 (61.1%)	46/81 (61.1%)	46/81 (61.1%)	1.000
Body mass index, kg/m^2^	26.3 ± 3.5	26.8 ± 3.6	25.9 ± 3.4	0.137
*Medicine history*				
Coronary artery disease	65/162 (40.1%)	35/81 (43.2%)	30/81 (37.0%)	0.423
Diabetes mellitus	45/162 (27.8%)	21/81 (25.9%)	24/81 (29.6%)	0.599
Hyperlipidemia	94/162 (58.0%)	50/81 (61.7%)	44/81 (54.4%)	0.339
Systolic blood pressure, mmHg	135.8 ± 17.1	136.4 ± 15.7	135.1 ± 18.5	0.622
Diastolic blood pressure, mmHg	76.7 ± 12.6	75.0 ± 12.0	78.4 ± 13.2	0.083
Mean arterial pressure, mmHg	96.4 ± 12.4	95.5 ± 11.8	97.3 ± 13.0	0.345
Heart rate, bpm	77.3 ± 13.4	74.2 ± 11.7	80.3 ± 14.3	0.004**
*Laboratory data*				
BNP level, pg/ml	54.0 (21.0,131.0)	26.5 (11.0,53.3)	106 (56.0,183.5)	< 0.001***
Creatine level, umol/l	69.7 (59.5,79.9)	69.4 (59.3,79.7)	71.1 (59.3,81.4)	0.707
Hemoglobin A1C, %	6.2 ± 0.9	6.2 ± 0.8	6.2 ± 1.0	0.868
LDL-c, mmol/l	2.3 ± 0.8	2.4 ± 0.9	2.2 ± 0.7	0.252
HDL-c, mmol/l	1.1 ± 0.3	1.1 ± 0.2	1.0 ± 0.3	0.465
Total cholesterol, mmol/l	4.2 ± 1.0	4.3 ± 1.0	4.0 ± 0.9	0.093
Homocysteine, umol/l	16.2 ± 6.4	15.7 ± 5.5	16.7 ± 7.2	0.441
ESR, mm/h	5.0 (2.0,10.0)	5.0 (2.0,11.0)	5.0 (2.0,9.0)	0.553
Hs-CRP, mg/L	1.5 (0.7,2.9)	1.4 (0.7,2.8)	1.6 (0.7,3.4)	0.741
Troponin I, ng/mL	0.00 (0.00,0.01)	0.00 (0.00,0.01)	0.01 (0.00,0.02)	0.094
ELABELA, ng/mL	3.1 (2.0,4.3)	4.0 (3.4,5.0)	2.0 (1.5,2.8)	< 0.001***
*Echocardiographic data*				
LAD, mm	39.8 ± 5.9	36.6 ± 5.3	42.6 ± 6.6	< 0.001***
LVEDd, mm	47.0 ± 4.1	47.1 ± 3.7	46.9 ± 4.5	0.802
LVEDs, mm	29.4 ± 4.9	29.0 ± 4.7	29.7 ± 5.0	0.427
LVEF, %	64.8 ± 7.6	66.2 ± 7.2	63.5 ± 8.1	0.038*
*Antihypertensive medication*				
ACEI or ARB	88/162 (54.3%)	45/81 (55.6%)	43/81 (53.1%)	0.752
Beta blocker	76/162 (46.9%)	38/81 (46.9%)	38/81 (46.9%)	1.000
CCBs	72/162 (44.4%)	37/81 (45.7%)	35/81 (43.2%)	0.752
Diuretics	19/162 (11.7%)	8/81 (9.9%)	11/81 (13.6%)	0.464

*BNP* brain natriuretic peptide, *LDL-c* low density lipoprotein cholesterol, *HDL-c* high density lipoprotein cholesterol, *ESR* erythrocyte sedimentation rate, *hs-CRP* high-sensitivity C-reactive protein, *LAD* left atrial diameter, *LVEDd* left ventricular end diastolic diameter, *LVEDs* left ventricular end systolic diameter, *LVEF* left ventricular ejection fraction, *ACEI* angiotensin-converting enzyme inhibitor, *ARB* angiotensin receptor antagonists, *CCBs* Calcium channel blockers

^*^P value less than 0.05; **P value less than 0.01; ***P value less than 0.001

The results of the correlation analyses are displayed in Table 2. ELABELA was negatively related to age (r =  −0.300, P < 0.001), HR (r =  −0.156, P = 0.047), BNP levels (r =  −0.330, P < 0.001) and LAD (r =  −0.289, P = 0.001). We further investigated the clinical characteristics associated with AF, which may be the underlying risk factor for AF development in patients with hypertension. In univariate analysis, heart rate, BNP levels, total cholesterol levels, ELABELA plasma levels and LAD were included in the equation (P < 0.1). Finally, ELABELA plasma levels (OR 0.018, 95% CI 0.029–0.224, P < 0.001) and LAD (OR 1.273, 95% CIL 1.102–1.472, P = 0.001) had statistical significance in multiple analyses (Table 3).

**Table 2:** Correlation between ELABELA and study variables in all subjects

	r	P value
Age, years	− 0.300	< 0.001***
Male sex	0.007	0.932
Body mass index, kg/m^2^	0.158	0.074
Coronary heart disease	0.007	0.926
Diabetes Mellitus	− 0.016	0.840
Hyperlipidemia	0.107	0.174
Systolic blood pressure, mmHg	0.122	0.121
Diastolic blood pressure, mmHg	− 0.010	0.900
Mean arterial pressure, mmHg	0.052	0.513
Heart rate, bpm	− 0.156	0.047*
BNP, pg/ml	− 0.330	< 0.001***
Creatine, umol/l	− 0.134	0.110
Hemoglobin A1C, %	− 0.040	0.648
LDL-c, mmol/l	0.076	0.369
HDL-c, mmo/l	0.084	0.327
Total cholesterol, mmol/l	0.130	0.125
Homocysteine, umol/l	− 0.134	0.113
ESR, mm/h	− 0.073	0.417
Hs-CRP, mg/l	− 0.152	0.100
Troponin I, ng/ml	− 0.010	0.908
LAD, mm	− 0.289	0.001**
LVEDd, mm	0.003	0.976
LVEDs, mm	− 0.089	0.326
LVEF, mm	0.117	0.195
ACEI or ARB	0.096	0.223
Beta blocker	0.052	0.512
CCBs	− 0.046	0.560
Diuretics	− 0.068	0.390

*BNP* brain natriuretic peptide, *LDL-c* low density lipoprotein cholesterol, *HDL-c* high density lipoprotein cholesterol, *ESR* erythrocyte sedimentation rate, *hs-CRP* high-sensitivity C-reactive protein, *LAD* left atrial diameter, *LVEDd* left ventricular end diastolic diameter, *LVEDs* left ventricular end systolic diameter, *LVEF* left ventricular ejection fraction, *ACEI* angiotensin-converting enzyme inhibitor, *ARB* angiotensin receptor antagonists, *CCBs* Calcium channel blockers

^*^P value less than 0.05; **P value less than 0.01; ***P value less than 0.001

**Table 3:** Clinical characteristics associated with AF in patients with hypertension

	Univariate analysis		Multivariate analysis	
	OR (95% CI)	P value	OR (95% CI)	P value
HR	1.037 (1.011–1.063)	0.005**		
BNP levels, pg/ml	1.010 (1.005–1.016)	< 0.001***		
Total cholesterol, mmol/l	0.744 (0.525–1.053)	0.095		
ELABELA levels, ng/ml	0.125 (0.067–0.235)	< 0.001***	0.081 (0.029–0.224)	< 0.001***
LAD, mm	1.200 (1.111–1.296)	< 0.001***	1.273 (1.102–1.472)	0.001**

*BNP* brain natriuretic peptide, *LAD* left atrial diameter

^*^P value less than 0.05; **P value less than 0.01; ***P value less than 0.001

### Comparison of the characteristics between paroxysmal AF and persistent AF

We further examined the ELABELA levels in the paroxysmal and persistent AF subgroups. Baseline characteristics were depicted in Additional file 1: Table S1. There were no significant differences in age, sex ratios, BMI, medical history or blood pressure between the two groups. Patients in the paroxysmal AF group had a lower heart rate than those in the persistent AF group (74.2 ± 12.5 vs. 88.0 ± 12.6 bpm, P = 0.001). Most laboratory parameters had no significant differences between the two groups, except for the BNP, ESR and ELABELA levels. Patients with paroxysmal AF had lower BNP levels than in patients with persistent AF (77.0 [50.0, 131.0] vs. 160 [86.8, 243.3] pg/ml, P = 0.005), while the median ELABELA levels in the paroxysmal AF group were significantly higher than those in the persistent AF group (2.2 [1.8, 3.0] vs. 1.8 [1.4, 2.5] ng/ml, P = 0.012) (Fig. 1b). The mean level of ESR was higher in the persistent AF group than in the paroxysmal AF group (5.0 [3.0–11.5] vs. 3.0 [2.0–7.0], p = 0.049). Significant differences were also observed between the paroxysmal AF and persistent AF groups in LAD (40.1 ± 6.6 vs. 45.6 ± 5.3 mm, P < 0.001). The correlation between ELABELA and the study variables in AF patients was portrayed in Additional file 2: Table S2. ELABELA plasma levels were negatively related to HR (r =  −0.289, P = 0.009), BNP levels (r =  −0.278, P = 0.021) and ESR (r =  −0.284, P = 0.029), while there was no significant correlation between ELABELA and LAD. We compared the demographic and clinical characteristics of all AF patients to explore factors associated with persistent AF (Additional file 3: Table S3). Univariate logistic regression indicated that HR, BNP levels, high-density lipoprotein cholesterol levels, ELABELA levels and LAD were associated with persistent AF in patients with hypertension and AF (P < 0.10). Multivariate analysis signalled that only HR (OR 1.133, 95% CI 1.052–1.220, P = 0.001) and LAD (OR 1.115, 95% CI 1.034–1.289, P = 0.011) were associated with persistent AF.

## Discussion

ELABELA, a novel endogenous ligand of APJ and a new member of the apelinergic system, was first detected in zebrafish embryos in 2013 [8]. The ELABELA gene is located on chromosome 4, and ELABELA-32 is its mature secretory form [13]. In addition to promoting heart development, accumulating evidence suggests that ELABELA plays important roles in the cardiovascular system in adults, and is closely linked to some heart diseases that are similar to apelin, another well-known ligand of APJ [14, 15]. The effects of ELABELA on the cardiovascular system include hypotensive effects, positive inotropic action, diuresis, anti-inflammatory, anti-oxidative stress, anti-fibrotic and anti-remodelling effects. The correlation between the plasma levels of ELABELA and patients with heart diseases, including hypertension and myocardial infarction, demonstrated that the protective effects of ELABELA existed not only in animal studies, but also in clinical studies [9, 16]. It is well established that these protective effects of ELABELA can either combat hypertension or prevent the development of AF. On the other hand, evidence from previous research indicates that apelin has a close relationship with AF [17, 18]. Given the underlying interaction among hypertension, AF and ELABELA, it is necessary to investigate the plasma levels of ELABELA in hypertensive people with and without AF.

This is the first study to date reporting that ELABELA plasma levels were lower in hypertensive patients with AF than in those without AF. We also found that ELABELA showed a close relationship with age, heart rate, BNP level and LAD. The subgroup analysis revealed that ELABELA levels were lower in the persistent AF group than in the paroxysmal AF group. These results imply that ELABELA, as a promising biomarker for hypertensive populations with AF, needs to be further investigated. According to current evidence and our findings, we proposed a hypothesis that insufficient ELABELA may partly contribute to AF development in the hypertensive population and could be a potential therapeutic target.

Both hypertension and AF are age-related diseases. Increasing age and organ ageing play a tremendous role in the development of these ailments [19]. The apelin/APJ system directly participates in the negative regulation of senescence-associated proteins, including P16, P21 and P53 [20]. The apelin/APJ system also alleviates the activation of the renin-angiotensin system (RAS), oxidative stress and inflammation, which are recognised inducers of senescence [21]. ELABELA, as a new member of the apelinergic system, may have a close relationship with age and exert anti-ageing effects. In our study, the ELABELA plasma level was negatively correlated with age, which supports the hypothesis above. Although there is no study providing direct evidence on the anti-ageing effects of ELABELA, ELABELA suppresses the expression of P53 in different scenarios [22, 23]. Both hypertension and AF progression are associated with cell senescence burden, as determined by p53 [24, 25]. Hence, ELEBELA may suppress these age-related illnesses in a P53-dependent manner. Further, ELABELA can antagonise the effects of angiotensin II, a classic senescence inducer [26]. These underlying anti-ageing mechanisms of ELABELA may be the bridge connecting decreased ELABELA plasma levels and hypertensive patients with AF.

The negative relationship between ELABELA and BNP may be attributed to the positive inotropic action and hypotensive effects of ELABELA [27]. It has been well established that impaired cardiac systolic function leads to arterial hypertension, which is a key pathophysiological change in AF development [28]. Decreased ELABELA plasma levels affect cardiac systolic function and elevate atrial pressure to increase AF incidence. On the other hand, insufficient anti-hypertensive effects also contribute to atrial remodelling and AF development. We also observed that the left atrium was enlarged in hypertension patients with AF and negatively related to ELABELA levels. This result can be interpreted by the multiple effects of ELABELA on anti-remodelling, which is the most important pathologic change in the enlarged left atrium and substrate of AF [9]. ELABELA has been illustrated to directly suppress hypertension, RAS activation, oxidative stress and chronic low-grade inflammation, which all contribute to atrial remodelling [29]. The diverse effects of ELABELA/APJ are derived from the activation of different downstream signalling pathways, including the inhibition of PI3K/Akt/mTOR, TGF-β1, FoxM1 and the expression of fibrosis-associated genes (*factor-β, latent TGFβ-binding protein 2, periostin and collagen 8a*). Accordingly, there are reasons to believe that the decreased ELABELA plasma level may not only be an associated factor of AF, but also serve as a novel risk factor for AF. ELABELA may become a promising biomarker in identifying hypertensive patients who are at high risk of developing AF or even an intervention target for AF management in the future.

Hypertension is associated with the activation of the renin-angiotensin system, and elevated angiotensin II has been linked to AF [30]. Angiotensin II is not only a senescence inducer but also an important pathogenesis for hypertension with AF [31]; this induces oxidative stress, inflammatory effects, apoptosis, necrosis and fibrosis. The potent and diverse effects of angiotensin II make it a core factor in the pathogenesis of hypertension with AF. On the other hand, blocking the renin-angiotensin system can effectively prevent AF development [30]. ELABELA has been shown to antagonise the renin-angiotensin system and to alleviate angiotensin II-induced cardiac damage [26, 32]. Further research has revealed that ELABELA suppresses the expression of angiotensin converting enzyme, an important enzyme that helps to generate angiotensin II [26]. Although the mechanism of interaction between ELABELA and the renin-angiotensin system is still not well known, ELABELA inhibits the renin-angiotensin system and may become a novel therapeutic target for hypertension and AF.

Another finding in this study is that ELABELA plasma levels were different in hypertensive patients with different types of AF. Patients with persistent AF had lower ELABELA levels than those with paroxysmal AF. This outcome indicates that ELABELA may also play a role in the pathological process of AF, as these two types of AF may be in different phases during disease progression [31]. Lower ELABELA plasma levels were present in patients with persistent AF who had a larger atrial size and higher BNP levels in our study. Enlarged atria is a crucial risk factor for the maintenance of AF [31]. Myocardial fibrosis and inflammation are two other vital AF substrates, and the severity of these pathological changes is associated with persistent AF [33]. Interestingly, ELABELA can inhibit myocardial fibrosis, suppress inflammatory effects, reverse atrial remodelling and may finally reduce the AF burden [34, 35]. We also found that ELABELA has a negative relationship with ESR, a traditional marker of inflammation. This outcome supports the viewpoint that ELABELA may reduce the AF burden by depressing inflammation [35]. Although the decreased plasma levels of ELABELA had a close relationship with AF in our study, they did not correlate with persistent AF in our subgroup analysis. ELABELA might not be a confounding factor but rather a critical factor in identifying AF types. The effect of the subtle differences in ELABELA plasma levels between patients with paroxysmal and persistent AF may be diminished when significant differences in atrial size are considered together. Notably, this result was based on subgroup analysis with a limited sample size. Whether a decreased ELABELA level is an important underlying risk factor for the maintenance of AF needs further well-designed studies to provide a clear answer.

## Limitations

This study is a monocentric study, which may induce selection bias. The small sample size may have reduced the reliability of the subgroup analysis.

## Conclusion

Our study revealed that plasma ELABELA levels are lower in hypertension patients with AF, and further decrease in persistent AF patients compared to paroxysmal AF patients. ELABELA plasma levels are negatively associated with age, BNP and LAD and may become a novel biomarker for AF in hypertensive populations.

## Supplementary Information

**Additional file 1. Table S1**: Comparison of the Demographic and Baseline Characteristics of the Paroxysmal AF and Persistent AF Groups.

**Additional file 2. Table S2**: Correlation between ELABELA and Study Variables in AF Patients.

**Additional file 3. Table S3**: Clinical Characteristics Associated with Persistent AF in AF Patients.

## Data Availability

The data used to support the findings of this study are available from the corresponding author upon request. The data will not be shared because they will be used for follow-up studies.

## Abbreviations

AF: Atrial fibrillation
eGFR: Estimated glomerular filtration rate
BNP: Brain natriuretic peptide
ESR: Erythrocyte sedimentation rate
LAD: Left atrial diameter
RAS: Renin-angiotensin system
