# Supplementary material for: Declined ELABELA plasma levels in hypertension patients with atrial fibrillation: a case control study
Source: BMC Cardiovasc Disord. 2021 Aug 12;21:390. doi: 10.1186/s12872-021-02197-x (PMC8359615; doi:10.1186/s12872-021-02197-x)
Supplement: Supplementary file 1 — Additional file 1. Table S1: Comparison of the Demographic and Baseline Characteristics of the Paroxysmal AF and Persistent AF Groups. [file 12872_2021_2197_MOESM1_ESM.docx]

**Supplementary Table 1.** Comparison of the Demographic and Baseline Characteristics of the Paroxysmal AF and Persistent AF Groups

|  | Paroxysmal AF group  (n=45) | Persistent AF group  (n=36) | P-value |
| --- | --- | --- | --- |
| Age, years | 66.9±9.3 | 66.9±11.2 | 0.992 |
| Male sex | 24/45(53.3%) | 22/36(61.1%) | 0.483 |
| Body mass index, kg/m^2^ | 25.8±3.6 | 25.9±3.2 | 0.888 |
| **Medicine History** |  |  |  |
| Coronary artery disease | 20/45(44.4%) | 10/36(27.8%) | 0.123 |
| Diabetes Mellitus | 11/45(24.4%) | 13/36(36.1%) | 0.253 |
| Hyperlipidemia | 28/45(62.2%) | 16/36(44.4%) | 0.110 |
| Systolic blood pressure, mmHg | 136.8±18.5 | 133.0±18.6 | 0.360 |
| Diastolic blood pressure, mmHg | 77.1±11.5 | 80.0±15.1 | 0.338 |
| Mean arterial pressure, mmHg | 97.0±11.5 | 97.7±14.9 | 0.834 |
| Heart rate, bpm | 74.2±12.5 | 88.0±12.6 | 0.001** |
| **Laboratory data** |  |  |  |
| BNP level, pg/ml | 77.0(50.0,131.0) | 160(86.8,243.3) | 0.005** |
| Creatine level, umol/l | 68.2(60.2,78.2) | 73.0(57.5,84.3) | 0.357 |
| Hemoglobin A1C, % | 6.2±1.1 | 6.2±0.9 | 0.748 |
| LDL-c, mmol/l | 2.2±0.8 | 2.3±0.6 | 0.588 |
| HDL-c, mmo/l | 1.1±0.3 | 1.0±0.2 | 0.054 |
| Total cholesterol, mmol/l | 4.0±1.0 | 4.0±0.8 | 0.934 |
| Homocysteine, umol/l | 16.7±7.2 | 16.9±7.4 | 0.867 |
| ESR mm/h | 3.0(2.0-7.0) | 5.0(3.0-11.5) | 0.049* |
| Hs-CRP, mg/L | 1.6 (0.7,3.4) | 1.6(0.7,3.3) | 0.863 |
| Troponin I, ng/mL | 0.01(0.00,0.02) | 0.01 (0.00,0.02) | 0.952 |
| ELABELA, ng/mL | 2.2(1.8,3.0) | 1.8(1.4,2.5) | 0.012* |
| **Echocardiographic data** |  |  |  |
| LAD, mm | 40.1±6.6 | 45.6±5.3 | <0.001*** |
| LVEDd, mm | 46.6±5.0 | 47.2±4.0 | 0.622 |
| LVEDs, mm | 29.6±4.8 | 30.0±5.4 | 0.743 |
| LVEF, % | 63.7±6.9 | 63.2±9.4 | 0.815 |
| **Medical therapy** |  |  |  |
| ACEI or ARB | 23/45(51.1%) | 20/36(55.6%) | 0.690 |
| Beta blocker | 18/45(40.0%) | 20/36(55.6%) | 0.163 |
| CCBs | 19/45(42.2%) | 16/36(44.4%) | 0.841 |
| Antiarrhythmic drug (class I) | 3/45(6.7%) | 0/36(0%) | 0.114 |
| Antiarrhythmic drug (class III) | 4/45(8.9%) | 0/36(0%) | 0.067 |
| Statins | 28/45(62.2%) | 19/36(52.8%) | 0.392 |
| Oral anticoagulants | 31/45(68.9%) | 27/36(75.0%) | 0.544 |

BNP, brain natriuretic peptide; LDL-c, low density lipoprotein cholesterol; HDL-c, high density lipoprotein cholesterol; ESR, erythrocyte sedimentation rate; hs-CRP, high-sensitivity C-reactive protein; LAD, left atrial diameter; LVEDd, left ventricular end diastolic diameter; LVEDs, left ventricular end systolic diameter; LVEF, left ventricular ejection fraction; ACEI, angiotensin converting enzyme inhibitor; ARB, angiotensin receptor blocker; CCBs, calcium channel blockers. * represents P value less than 0.05; **represents P value less than 0.01; *** represents P value less than 0.001.
