# Supplementary material for: Declined ELABELA plasma levels in hypertension patients with atrial fibrillation: a case control study
Source: BMC Cardiovasc Disord. 2021 Aug 12;21:390. doi: 10.1186/s12872-021-02197-x (PMC8359615; doi:10.1186/s12872-021-02197-x)
Supplement: Supplementary file 2 — Additional file 2. Table S2: Correlation between ELABELA and Study Variables in AF Patients. [file 12872_2021_2197_MOESM2_ESM.docx]

**Supplementary Table 2.** Correlation between ELABELA and Study Variables in AF Patients

|  | r | P value |
| --- | --- | --- |
| Age, years | -0.153 | 0.173 |
| Male sex | -0.124 | 0.271 |
| Body mass index, kg/m^2^ | 0.042 | 0.742 |
| Coronary heart disease | 0.106 | 0.346 |
| Diabetes Mellitus | 0.021 | 0.854 |
| Hyperlipidemia | 0.061 | 0.586 |
| Systolic blood pressure, mmHg | 0.100 | 0.375 |
| Diastolic blood pressure, mmHg | 0.034 | 0.765 |
| Mean arterial pressure, mmHg | 0.078 | 0.486 |
| Heart rate, bpm | -0.289 | 0.009** |
| BNP, pg/ml | -0.278 | 0.021* |
| Creatine, umol/l | -0.100 | 0.405 |
| Hemoglobin A1C, % | -0.177 | 0.161 |
| LDL-c, mmol/l | -0.131 | 0.285 |
| HDL-c, mmol/l | 0.219 | 0.070 |
| Total cholesterol, mmol/l | -0.111 | 0.366 |
| Homocysteine, umol/l | -0.083 | 0.550 |
| ESR, mm/h | -0.284 | 0.029* |
| Hs-CRP, mg/L | -0.211 | 0.126 |
| Troponin I, ng/ml | -0.089 | 0.473 |
| LAD, mm | -0.038 | 0.726 |
| LVEDd, mm | 0.155 | 0.210 |
| LVEDs, mm | 0.053 | 0.669 |
| LVEF, mm | -0.103 | 0.407 |

BNP, brain natriuretic peptide; LDL-c, low density lipoprotein cholesterol; HDL-c, high density lipoprotein cholesterol; ESR, erythrocyte sedimentation rate; hs-CRP, high-sensitivity C-reactive protein; LAD, left atrial diameter; LVEDd, left ventricular end diastolic diameter; LVEDs, left ventricular end systolic diameter; LVEF, left ventricular ejection fraction. * represents P value less than 0.05; ** represents P value less than 0.01.
