# Supplementary material for: Declined ELABELA plasma levels in hypertension patients with atrial fibrillation: a case control study
Source: BMC Cardiovasc Disord. 2021 Aug 12;21:390. doi: 10.1186/s12872-021-02197-x (PMC8359615; doi:10.1186/s12872-021-02197-x)
Supplement: Supplementary file 3 — Additional file 3. Table S3: Clinical Characteristics Associated with Persistent AF in AF Patients. [file 12872_2021_2197_MOESM3_ESM.docx]

**Supplementary Table 3.** Clinical Characteristics Associated with Persistent AF in AF Patients

|  | Univariate analysis | | Multivariate analysis | |
| --- | --- | --- | --- | --- |
|  | OR(95% CI) | P value | OR(95% CI) | P value |
| HR, bpm | 1.091  (1.044-1.141) | <0.001 | 1.133  (1.052-1.220) | 0.001** |
| BNP levels, pg/ml | 1.007  (1.001-1.013) | 0.015 |  |  |
| HDL-c, mg/dl | 0.168  (0.026-1.078) | 0.060 |  |  |
| ELABELA levels, ng/ml | 0.548  (0.308-0.977) | 0.042 |  |  |
| LAD, mm | 1.161  (1.059-1.273) | 0.001 | 1.115  (1.034-1.289) | 0.011* |

BNP, brain natriuretic peptide; HDL-c, high density lipoprotein cholesterol; LAD, left atrial diameter. * represents P value less than 0.05; ** represents P value less than 0.01.
